# Supplementary material for: Predicting visual function by interpreting a neuronal wiring diagram
Source: Nature. 2024 Oct 2;634(8032):113–23. doi: 10.1038/s41586-024-07953-5 (PMC11446822; doi:10.1038/s41586-024-07953-5)
Supplement: Supplementary file 6 — CRF and ERF predictions for individual TmY4 and TmY9 cells. Analogous to Supplementary Data 3, but for TmY target types. Shown are the top four monosynaptic pathways, the strongest pathway passing through each of the top ten intermediary types (ranking from Extended Data Fig. 7), and the trisynaptic pathway Tm1–TmY–Dm3–TmY (see the section entitled Prediction of spatial normalization). [file 41586_2024_7953_MOESM6_ESM.zip › DataS4/TmY9qΓèÑ/720575940637728996.pdf]

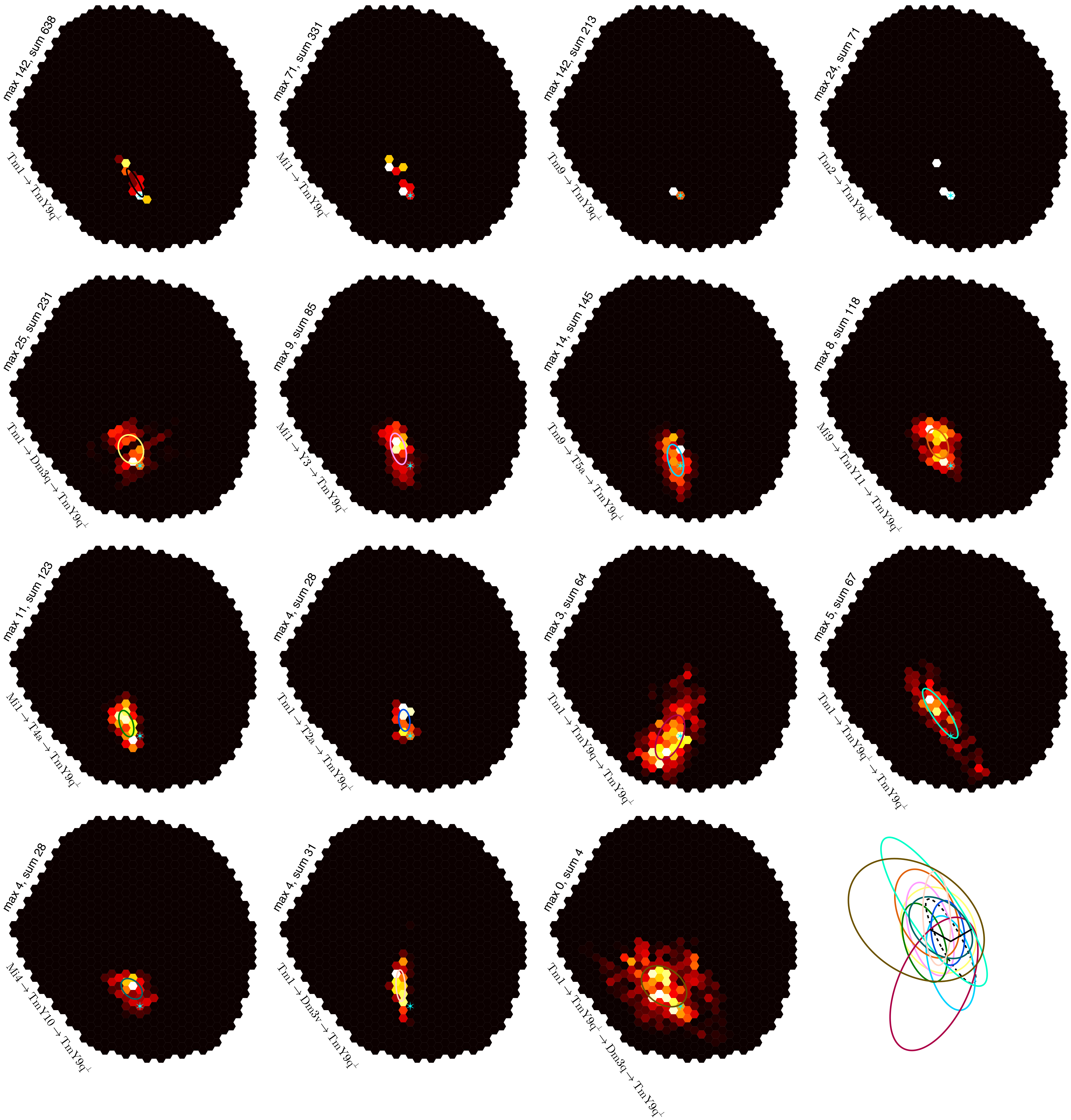

max 142, sum 638
Tm1 → TmY9q⊥
max 71, sum 331
Mi1 → TmY9q⊥
max 142, sum 213
Tm9 → TmY9q⊥
max 24, sum 71
Tm2 → TmY9q⊥
max 25, sum 231
Tm1 → Dm3q → TmY9q⊥
max 9, sum 85
Mi1 → Y3 → TmY9q⊥
max 14, sum 145
Tm9 → T5a → TmY9q⊥
max 8, sum 118
Mi9 → TmY11 → TmY9q⊥
max 11, sum 123
Mi1 → T4a → TmY9q⊥
max 4, sum 28
Tm1 → T2a → TmY9q⊥
max 3, sum 64
Tm1 → TmY9q → TmY9q⊥
max 5, sum 67
Tm1 → TmY9q⊥ → TmY9q⊥
max 4, sum 28
Mi4 → TmY10 → TmY9q⊥
max 4, sum 31
Tm1 → Dm3v → TmY9q⊥
max 0, sum 4
Tm1 → TmY9q⊥ → Dm3q → TmY9q⊥
